# Subarachnoid Hemorrhage Promotes Proliferation, Differentiation, and Migration of Neural Stem Cells via BDNF Upregulation

**DOI:** 10.1371/journal.pone.0165460

**Published:** 2016-11-10

**Authors:** Wen-Di Lee, Kuo-Chuan Wang, Yi-Fen Tsai, Pin-Chun Chou, Li-Kai Tsai, Chung-Liang Chien

**Affiliations:** 1 Graduate Institute of Anatomy and Cell Biology, College of Medicine, National Taiwan University, Taipei, Taiwan; 2 Department of Surgery, National Taiwan University Hospital and National Taiwan University College of Medicine, Taipei, Taiwan; 3 Department of Neurology and Stroke Center, National Taiwan University Hospital and National Taiwan University College of Medicine, Taipei, Taiwan; Temple University School of Medicine, UNITED STATES

## Abstract

Patients who suffer from subarachnoid hemorrhage (SAH) usually have long-term neurological impairments. Endogenous neurogenesis might play a potential role in functional recovery after SAH; however, the underlying neurogenesis mechanism is still unclear. We assessed the extent of neurogenesis in the subventricular zone (SVZ) to better understand the neurogenesis mechanism after SAH. We performed a rat model of SAH to examine the extent of neurogenesis in the SVZ and assessed functional effects of the neurotrophic factors in the cerebrospinal fluid (CSF) on neural stem cells (NSCs) after SAH. In this study, the proliferation, differentiation, and migratory capacities of NSCs in the SVZ were significantly increased on days 5 and 7 post SAH. Furthermore, treatment of cultured rat fetal NSCs with the CSF collected from rats on days 5 and 7 post SAH enhanced their proliferation, differentiation, and migration. Enzyme-linked immunosorbent assay (ELISA) of the CSF detected a marked increase in the concentration of brain-derived neurotrophic factor (BDNF). Treating the cultured NSCs with recombinant BDNF (at the same concentration as that in the CSF) or with CSF from SAH rats, directly, stimulated proliferation, differentiation, and migration to a similar extent. BDNF expression was upregulated in the SVZ of rats on days 5 and 7 post SAH, and BDNF release occurred from NSCs, astrocytes, and microglia in the SVZ. These results indicate that SAH triggers the expression of BDNF, which promotes the proliferation, differentiation, and migration of NSCs in the SVZ after SAH.

## Introduction

Subarachnoid hemorrhage (SAH) is a fatal type of stroke and survivors usually have long-term physical, neurocognitive, psychological, and/or psychiatric impairments [1]. Current standard management for SAH is mainly supportive and aimed at preventing complications. However, there is no definite treatment promoting neurological recovery. Stem cell therapy is an advanced stroke treatment that could potentially improve functional outcome [2]. Adult endogenous neural stem cells (NSCs) proliferate, differentiate, and migrate from the subventricular zone (SVZ) of the brain [3–5] to play major roles in neurological recovery after ischemic stroke [6, 7]. Therefore, investigating endogenous neurogenesis after SAH might suggest a future cell-based therapy for SAH.

Previous studies have noted the higher proliferation capacity of NSCs in the SVZ of rats with SAH and the activation of endogenous NSCs in the brains of adult humans with SAH [8, 9]. However, the characteristics and underlying mechanisms of endogenous neurogenesis in SAH are still unclear. The cerebrospinal fluid (CSF) functions as a sink for brain extracellular solutes that pass through the brain interstitial space [10]. Therefore, certain factors that trigger neurogenesis after SAH may be apparent and measurable in the CSF, affording the opportunity to find out the main factor contributing to endogenous neurogenesis in SAH. In the present investigation of endogenous neurogenesis in the SVZ after SAH, we used CSF analysis to identify brain-derived neurotrophic factor (BDNF) as the key factor associated with neurogenesis after SAH.

## Materials and Methods

### Ethics statement

This research program using the animal experiment protocols specified below was approved by the Utilization Committee and the National Taiwan University Institutional Laboratory Animal Care committee (Approval no.: IACUC-20140071). All procedures met the requirements of the Animal Welfare Protection Act of the Department of Agriculture, Executive Yuan, Taiwan. All surgery was performed under anesthesia by using 2.5% isoflurane with 70% nitrous oxide and 27.5% oxygen. Animals were sacrificed by using overdose of sodium pentobarbital and all efforts were made to minimize suffering.

### Animal model

Adult male Wistar rats weighing from 280 to 300 g were anesthetized by using 2.5% isoflurane with 70% nitrous oxide and 27.5% oxygen. A small suboccipital incision was made to expose the arch of the atlas, the occipital bone, and the atlantooccipital membrane overlying the cisterna magna. The cisterna magna was tapped using a U-100 insulin syringe with 28G x 1/2 inch needle (BD Biosciences, San Jose, CA), and 0.1 ml of CSF was then gently aspirated. The femoral artery was exposed and a PE-50 tube connected with 0.5-ml syringe was introduced into the artery. Approximately 0.2 ml of blood drawn from femoral artery was injected into the cisterna magna over a period of 2 to 3 minutes. In sham-operated controls, normal saline was injected into the cisterna magna. Immediately after the injection of blood, the hole was sealed with *Super Glue*® (Super Glue Corporation, Ontario, CA) to prevent fistula formation. After suturing the skin, rats were placed in a cage under an infrared heating lamp until recovery from anesthesia. The incision site was examined daily for evidence of wound dehiscence or infection until it is completely healed. In general, the animals showed drowsiness immediately after SAH induction. In rare cases mild paresis of hind limbs could be observed.

In this study 60 rats were totally used, including 44 male rats for SAH animal model (6 per each SAH time points, 12 rats for control and 8 rats were dead during the induction of SAH) and 16 female pregnant Wistar rats for primary NSC culture. The mortality rate was 25% and the animals were randomized to experimental conditions.

### Immunohistochemistry

Immunohistochemistry was performed on cryostat brain sections that were collected from rats 1, 3, 5, and 7 days after SAH induction and sham induction. Sections were permeabilized with 1% Triton X100-PBS, incubated with 3% fetal bovine serum (FBS) in 0.1% Triton X100-PBS at room temperature for 2 hours to block nonspecific binding, incubated with primary antibodies in blocking solution (3% fetal bovine serum in 0.1% triton X100-PBS) at 4°C overnight, incubated with secondary antibodies and Hoechst 33342 (1:1000 diluted in PBS; Life Technologies) for 1 hour at room temperature, and mounted with Fluoro-Gel (Electron Microscopy Sciences, Hatfield, PA). Detailed information of antibodies used in this experiment were provided in the supporting information (S1 Text). All images were acquired with a Leica TCS SP5 confocal microscope (Leica Microsystems).

### Primary culture of neural stem cells

Telencephalon of gestational age day 15 Wistar rat was dissected out. The telencephalon tissues were pooled in a 50-ml tube and centrifuged at 1000 rpm for 5 minutes. The tissue pellet was then dissociated into single cells by triturating through a 1000-μl pipette. Cultures were incubated at 37°C in a humidified atmosphere and 5% CO_2_ for 6 days by which time primary neurospheres had formed. Detailed procedure were provided in supporting information (S1 Text).

### Immunocytochemistry

Primary neurospheres were seeded on coverslips in wells of a 12-well plate containing complete medium with 0.5% CSF, and cultured for 24 hours with or without treatment with 0.5% CSF or 5 pg/ml BDNF. Cells were then fixed with 4% paraformaldehyde (PFA), incubated with 1% FBS in 1% Triton X100-PBS for 1 hour at room temperature to block nonspecific binding, incubated with primary antibodies diluted in blocking solution overnight at 4°C, incubated with secondary antibodies and Hoechst 33342 (diluted in blocking solution) for 1 hour at room temperature, and mounted on microscope slides (Marienfeld-Superior, Lauda-Königshofen, Germany) with Fluoro-Gel. Detailed information of antibodies used in this experiment were provided in the supporting information (S1 Text).

### Enzyme-Linked Immunosorbent Assay (ELISA)

The levels of erythropoietin (EPO), BDNF, beta-neural growth factor (NGF), and epidermal growth factor (EGF) protein were determined (detailed information of ELISA kits were provided in the supporting information [S1 Text]). All procedures were performed following the manual supplied by the manufacturer. After the reaction was terminated by stop solution, the optical density was read at 450 nm on a microplate reader (Amersham Bioscience, Buckinghamshire, UK).

### Quantification in images

The immunoreactivity for Ki67, nestin, BDNF, Iba-1, and ED-1 was determined in the dorsolateral SVZ and that for DCX and GFAP was determined in the lateral SVZ and striatum in 4 anatomically matched sections, 84 μm apart from each other, per animal. All experimental assessments were done in blind condition. The number of proliferating cells, BDNF-expressing cells, and microglia in the dorsolateral SVZ were semiquantitatively estimated using image analysis software. The resulting projection image was converted to grayscale and a similar threshold was set for all images and the area of specific immunoreactivity was measured using ImagePro Plus 6.0 (MediaCybernetics^®^). Immunoreactivity was then expressed as the total area of specific immunoreactivity within the total dorsolateral, lateral, and striatum areas. In images of the immunocytochemically stained neurospheres, neurospheres immunoreactive for Ki67 were counted. ImagePro Plus 6.0 was used to count the number of differentiated cells (i.e., cells immunopositive for DCX, GFAP, and Tuj-1 that migrated out from neurospheres). Detailed information of live cell imaging by time-lapse microscopy were provided in the supporting information (S1 Text).

### Statistical analysis

Values are expressed mean values ± SD. The image quantification and ELISA data were statistically processed using one way ANOVA followed by Tukey's multiple comparisons test or Dunnett's multiple comparisons test and plotted by GraphPadPrism^®^ 4.0 (GraphPad, La Jolla, CA). *P* <0.05 was considered statistically significant.

## Results

### SAH promotes NSC proliferation, differentiation, and migratory capacities in the SVZ

**Fig 1:**
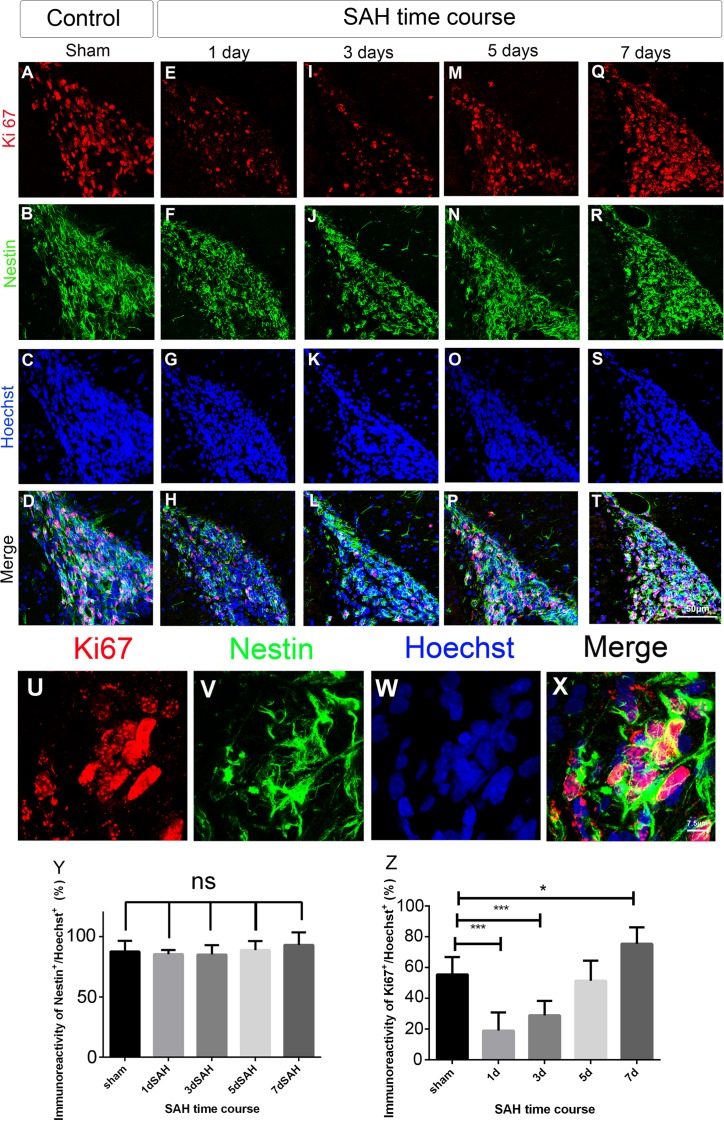
Cell proliferation at the SVZ in a rat model of SAH. Coronal sections of forebrain were immunostained with anti-Ki67 antibody (red, proliferating cell), anti-nestin antibody (green, NSC) and Hoechst 33342 (blue, nucleus).The level of cell proliferation in the SVZ was determined by Ki67/ Hoechst 33342 co-staining. (A)-(T): photomicrograph showing the distribution of Ki67+ (A, E, I, M, and Q) and nestin+ (B, F, J, N, and R) signals and as merged images (D, H, L, P, and T) in the SVZ of animals at different SAH time course (1, 3, 5, and 7 days after SAH) and sham control. Scale bar = 50 μm. (U)-(X): Images of Ki67+ /nestin+ NSCs in the SVZ on day 7 post SAH showing Ki67 (U) and nestin (V) immunoreactivity separately or as merged image (X). Scale bar = 7.5 μm. (Y): Percentage of NSCs (Nestin+ of Hoechst+ cells) in the SVZ. (Z): Percentage of proliferating cells (Ki67+ of Hoechst+ cells) in the SVZ. Means ± SD, n = 6 for each time-point. *, P < 0.05; ***, P < 0.001, one way ANOVA with Dunnett's multiple comparisons test.

To investigate the NSC proliferative capacity in the SVZ after SAH, forebrain sections from a rat model of SAH at different post-SAH times were immunostained with anti-Ki67 and anti-nestin antibodies. The cells in the SVZ were mainly positive for the NSC marker nestin (85–93%) and the percentage of these cells was similar in rats without SAH and rats with SAH at different times after SAH (Fig 1B, 1F, 1J, 1N, 1R and 1Y). Using Ki67 as a marker of proliferating cells, the percentage of Ki67-positive cells in the SVZ declined significantly below the baseline (sham control) level on post-SAH days 1 and 3 (19% and 28% *vs*. 55.5%, *P*< 0.001), returned to the baseline level (51.4%) on post-SAH day 5 (Fig 1A, 1E, 1I and 1Z), and increased significantly above the baseline on post-SAH day 7 (75%, *P* = 0.036; Fig 1M, 1P, 1Q, 1T and 1Z), which implied the enhancement of the proliferative capacity of NSCs in the SVZ 7 days after SAH.

**Fig 2:**
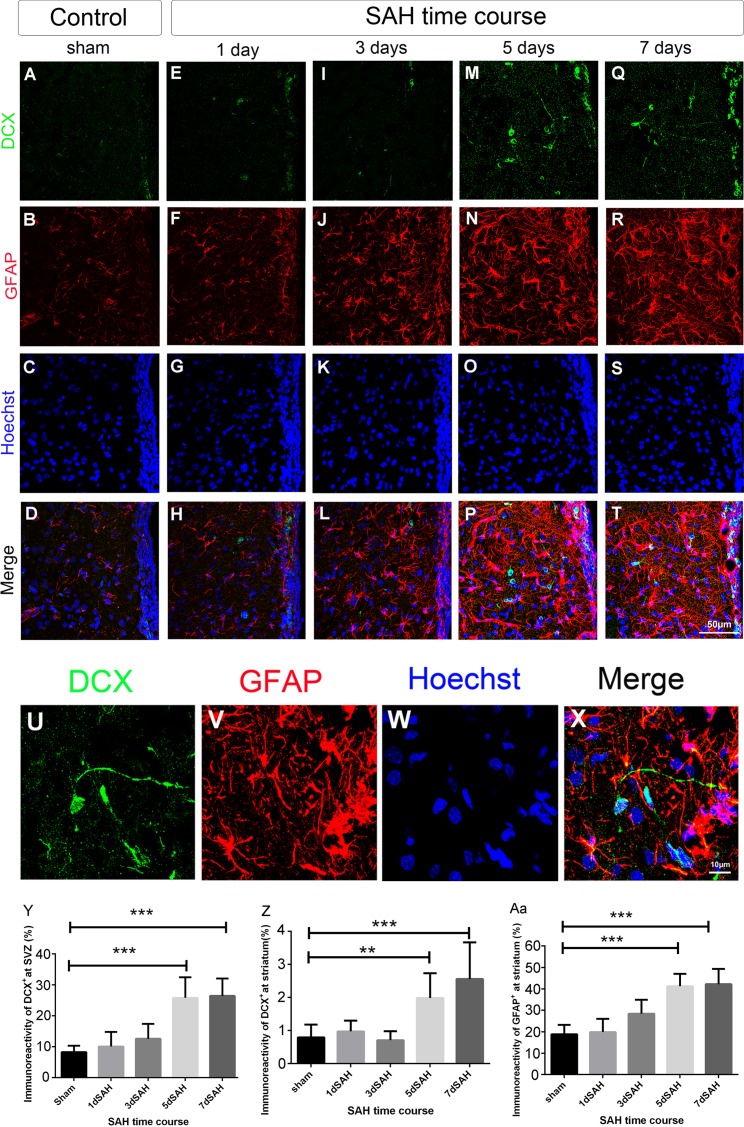
NSC differentiation and neuroblast migration around the SVZ after SAH. Coronal sections of forebrain were immunostained with anti-DCX (green: neuroblast), anti-GFAP (red: astrocyte) and Hoechst 33342 (blue: nucleus) to analyze the effect of SAH on NSC differentiation at the SVZ and neuroblast migration toward the striatum. (A)-(T): photomicrograph showing the distribution of DCX^+^ (A, E, I, M, and Q) and GFAP^+^ (B, F, J, N, and R) signals and as merged images (D, H, L, P, and T) at the SVZ of animals with different SAH time course (1, 3, 5, and 7 days after SAH) and sham control. Scale bar = 50 μm. (U)-(X): Images showing DCX^+^ (U) and GFAP^+^ (V) cells or as merged image (X) in the striatum on day 7 post SAH. Scale bar = 10 μm. (Y): Percentage of DCX^+^ area (DCX^+^ area/ total area) at the SVZ; (Z): Percentage of DCX^+^ area (DCX^+^ area/ total area) in the striatum 50 μm away from the SVZ; (Aa) Percentage of GFAP^+^ area (GFAP^+^ area/ total area) in the striatum. Means ± SD, n = 6 for each time-point. **, *P* < 0.01; ***, *P* < 0.001, one way ANOVA with Dunnett's multiple comparisons test.

We further used DCX (a neuroblast marker) and GFAP (an astrocyte marker) to determine the differentiation and migratory capacity of NSCs in the SVZ after SAH. DCX immunoreactivity in the SVZ was significantly above baseline (the sham control level) on 5 and 7 days post SAH (25.8% and 26% *vs*. 8%, *P* < 0.001) (Fig 2A, 2M, 2Q and 2Y), indicating enhancement of NSC differentiation into neural lineage cells after SAH. Previous studies have shown that in the nonstroke-derived brain slice adult SVZ cells located within the SVZ, in which SVZ cells do not migrate to the striatum; however, after stroke, SVZ cells migrated toward the ischemic striatum, indicating that in addition to increases in migratory capability, SVZ cells change their migratory direction after stroke.[11, 12] In our study, DCX immunoreactivity in the striatum (at least 50 μm away from the SVZ) was also significantly above baseline on 5 and 7 days post SAH (2% and 2.6% *vs*. 0.8%, *P* < 0.01) (Fig 2A, 2M, 2Q and 2Z), indicating promotion of the migratory capacity of neuroblasts after SAH. Moreover, striatal astrogliosis was profound on days 5 and 7 post SAH in contrast to the sham control (41% and 42% *vs*. 18%, *P* < 0.001) (Fig 2B, 2N, 2R and 2Aa). Taken together, the results indicate the induction of NSC differentiation (into neural and glial lineage cells) and migration of neuroblasts from the SVZ on days 5 and 7 post SAH. We also assess the ipsilateral cortex for the neuronal progenitor cells and astrocytes; however, little to none of them was found in this area (data not shown)

### Post-SAH CSF stimulates proliferation, differentiation, and migratory capacities of cultured neurospheres

Since the SVZ is near cerebroventricles without barriers and capable of releasing certain neurotrophic factors into the CSF after SAH, CSF was collected from SAH and control rats at different post-SAH time points (days 3, 5, and 7). CSF from rats at 7 days post SAH was first serially diluted to determine the optimal conditions for further analysis. Primary cultured neurospheres treated with CSF (CSF in cultured medium at 0.25, 0.5, 1 and 2% concentrations) for 24 hours were double immunostained with anti-nestin and Ki67 antibodies. The percentage of Ki67-positive cells was significantly higher at the concentrations of 0.5%, 1%, and 2% in SAH neurospheres than control neurospheres (48.4%, 48.8% and 50.7% *vs*. 21.7%, *P*< 0.001) (S1 Fig). The concentration of 0.5% CSF was thus applied for further *in vitro* studies.

**Fig 3:**
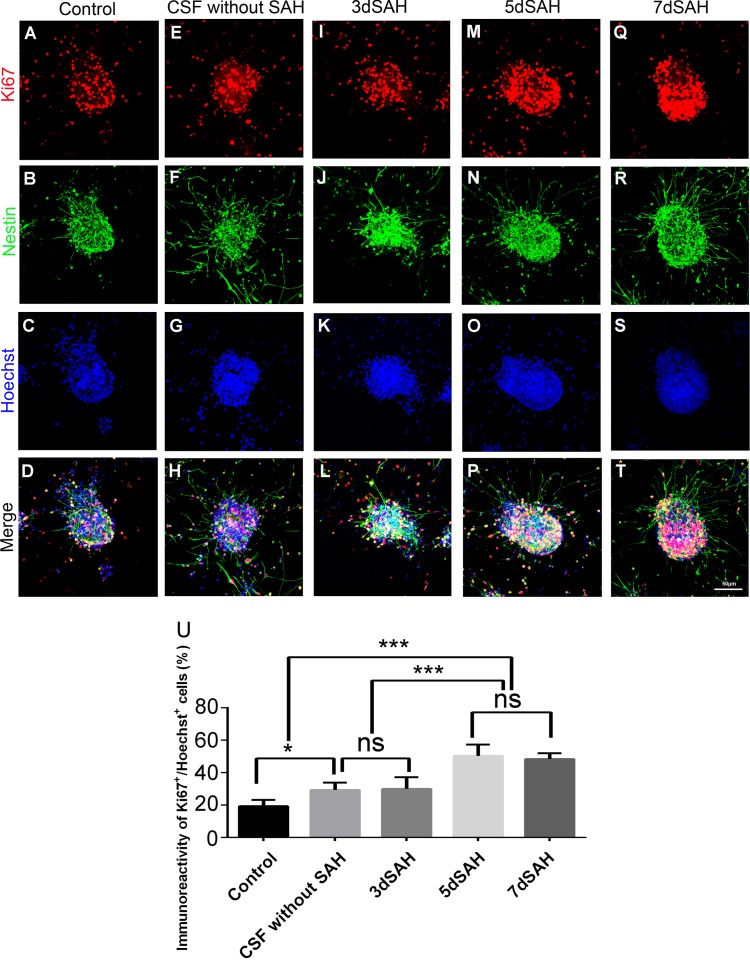
Treatment with post-SAH CSF promotes cell proliferation of cultured neurospheres. Cells were immunostained with anti-nestin antibody (green: NSC), Ki67 antibody (red: proliferating cell) and Hoechst 33342 (blue: nucleus) to determine the level of cell proliferation of cultured neurospheres. (A)-(T): photomicrograph showing the distribution of Ki67^+^ (A, E, I, M, and Q) and nestin^+^ (B, F, J, N, and R) signals and as merged images (D, H, L, P, and T) in the neurospheres treated with and without CSF. Scale bar = 50 μm. (U): Percentage of proliferating cells (Ki67^+^ of Hoechst^+^ cells) in the neurospheres. Means ± SD, 12 neurospheres were counted for each condition. ns: non-significant; *, *P* < 0.05; ***, *P* < 0.001, one way ANOVA with Tukey's multiple comparisons test.

The CSF collected from rats at different post SAH time points was then added to culture medium to see the effects of CSF on proliferation, differentiation, and migration of NSCs in cultured neurospheres. Almost all cells in neurospheres were positive for nestin (92~95%) in different groups (Fig 3B–3R). CSF collected 5 or 7 days post SAH in contrast to control CSF significantly increased the percentage of Ki67-positive cells in neurospheres (50.3% and 48.4% *vs*. 29.3%; *P*< 0.001), indicating post-SAH CSF could stimulate proliferation of cultured NSCs (Fig 3E, 3M, 3Q and 3U).

**Fig 4:**
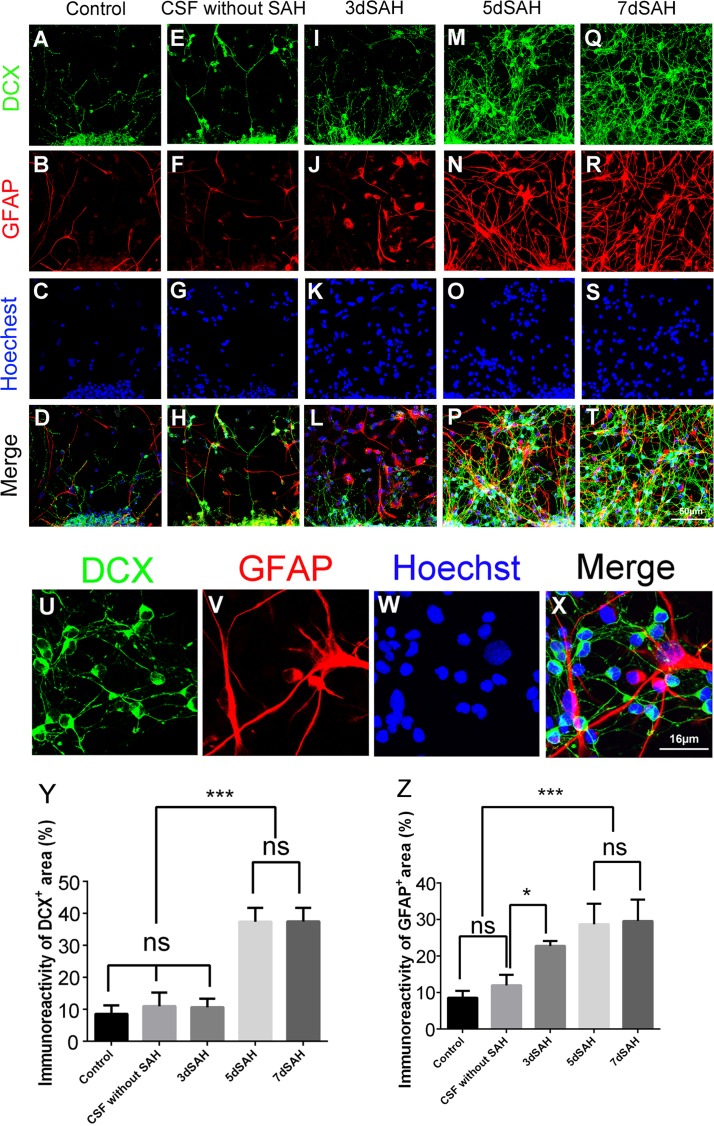
Treatment with post-SAH CSF enhances cell differentiation of cultured neurospheres. Cell differentiation were determined by immunocytostaining of cultured neurospheres with anti-DCX antibody (green: neuroblast), GFAP antibody (red: astrocyte) and Hoechst 33342 (blue: nucleus). (A)-(T): photomicrograph showing the distribution of DCX^+^ (A, E, I, M, and Q) and GFAP^+^ (B, F, J, N, and R) signals and as merged images (D, H, L, P, and T) in the neurospheres treated with and without CSF. Scale bar = 50 μm. (U)-(X): Images showing DCX^+^ (U) and GFAP^+^ (V) cells or as merged image (X) in the neurospheres treated with CSF collected from rats 7 days post SAH. Scale bar = 16 μm (Y): Percentage of DCX^+^ area (DCX^+^ area/ total area); (Z): Percentage of GFAP^+^ area (GFAP^+^ area/ total area). Means ± SD, 12 neurospheres were counted for each condition. ns: non-significant; *, *P* < 0.05; ***, *P* < 0.001, one way ANOVA with Tukey's multiple comparisons test.

CSF collected 5 or 7 days post SAH significantly increased the immunoreactivities of DCX and a mature neuronal marker, Tuj-1, over baseline (control) control values, respectively (DCX, 37.3% and 37.4% *vs*. 10.9%, *P*< 0.001; Tuj-1, 41.5% and 43.7% *vs*. 10.3%, *P*< 0.001), implying that post-SAH CSF can stimulate the neuronal differentiation of NSCs (Fig 4E, 4M, 4Q and 4Y and S2D, S2J, S2M and S2P Fig), and the immunoreactivity of GFAP over baseline (control) values (41.2% and 42.1% *vs*. 18.8%; *P*< 0.001), indicating post-SAH CSF could trigger astrocyte differentiation of NSCs (Fig 4F, 4N, 4R and 4Z). Time-lapse imaging of cell migration showed that CSF collected 7 days post SAH (S2 Movie) relative to untreated control (S1 Movie) stimulated the migratory capacity of NSCs around neurospheres, implying post-SAH CSF could stimulate NSC migration.

Taken together, these data indicate CSF collected on days 5 and 7 post SAH enhance the proliferation, differentiation (into neural and glial lineage cells), and migratory capacity of NSCs in cultured neurospheres.

### Increase of BDNF concentration in CSF from SAH rats

**Fig 5:**
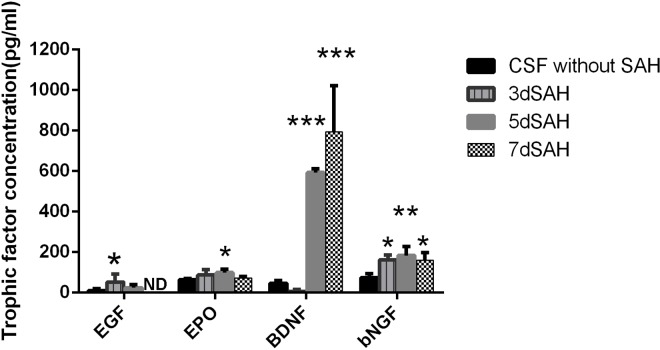
Concentration of trophic factors in CSF from SAH rats. ELISA analysis was applied to measure the concentration of trophic factors in the CSF. The concentration of BDNF was significantly increased in the CSF collected from rats on days 5 and 7 post SAH as compared to that in the CSF from rats without SAH. The concentration of bNGF, EPO and EGF was increased in the CSF from rats on day 3 or 5 post SAH. Results of ELISA were analyzed by one way ANOVA with Tukey's multiple comparisons test. *, *P* < 0.05; **, *P* < 0.01, ***, *P* < 0.001 compared with CSF without SAH. ND, non-detectable.

Enhanced expression of BDNF, EGF, EPO, and bNGF after cerebral ischemia or traumatic brain injuries may contribute to the activation of neurogenesis in the SVZ. We thus analyzed the concentrations of these neurotrophic factors in CSF collected before and after SAH using ELISA. The concentration of BDNF in CSF decreased relative to the baseline (control) value 3 days post SAH (5 *vs*. 46 pg/ml, *P* = 0.95) and increased markedly by 5 or 7 days post SAH (593 and 793 *vs*. 46 pg/ml, *P* <0.01) (Fig 5). On the other hand, the concentrations of EGF (52 *vs*. 10.8 pg/ml, *P* = 0.03), EPO (99 *vs*. 65 pg/ml, *P* = 0.02), and bNGF (183 *vs*. 74 pg/ml, *P* = 0.002) relative to their respective control values were modestly increased in CSF on day 3 or 5 post SAH (Fig 5), but not significantly increased on day 7 post SAH. Since the change in BDNF concentration paralleled changes in the proliferation, differentiation, and migration of NSCs both *in vivo* and *in vitro* (and all peaking on days 5 and 7 post SAH), we proposed that BDNF might be the major trophic factor stimulating neurogenesis of NSCs after SAH.

### Recombinant BDNF facilitates the proliferation, differentiation, and migratory capacities of NSCs in cultured neurospheres

**Fig 6:**
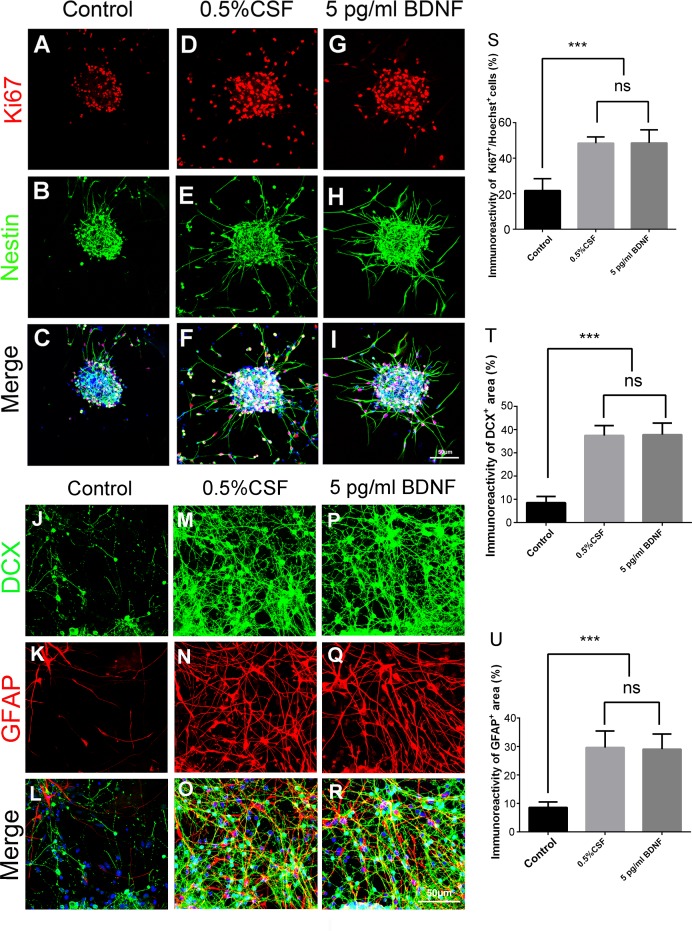
BDNF promotes cell proliferation, differentiation, and migration of cultured neurospheres. Neurospheres were immunostained with Ki67, nestin, DCX and GFAP antibodies to determine the effects of recombinant BDNF (5 pg/ml) on neurogenesis of cultured neurospheres. The groups of neurospheres treated with 0.5% CSF (collected from rats on day 7 post SAH) and without treatment (control) were used for comparison. (A)-(I): photomicrograph showing the distribution of Ki67^+^ (A, D, and G) and nestin+ (B, E, and H) signals and as merged images (C, F, and I); (J)-(R): photomicrograph showing the distribution of DCX^+^ (J, M, and P) and GFAP^+^ (K, N, and Q) signals and as merged images (L, O and R). Scale bar = 50 μm. (S): Percentage of proliferating cells (Ki67^+^ of Hoechst^+^ cells) in the neurospheres. (T): Percentage of DCX^+^ area (DCX^+^ area/ total area). (U): Percentage of GFAP^+^ area (GFAP^+^ area/ total area). Means ± SD, 12 neurospheres were counted for each condition. ns: non-significant; ***, P < 0.001, one way ANOVA with Tukey's multiple comparisons test.

To determine the effect of BDNF on neurogenesis, primary cultured neurospheres were treated with recombinant human BDNF at 5 pg/ml, which was about the 0.5% concentration of BDNF determined by ELISA in CSF on 7 days post SAH. The percentage of Ki67-positive cells was significantly higher in BDNF-treated neurospheres than in untreated neurospheres (48.5% *vs*. 21.7%, *P* <0.001), and was comparable to that (48.4%) in group of 0.5% CSF (CSF collected on 7 days post SAH) (Fig 6D, 6G and 6S) (CSF not exposed to SAH has little neurotrophic effects as untreated control shown in both Figs 3 and 4). BDNF treatment (relative to the control) increased the immunoreactivities of both DCX and Tuj-1 around neurospheres (DCX, 37.8% *vs*. 8.5%, *P* <0.001; Tuj-1, 38.6% *vs*. 9.9%, *P* <0.001) and the increase was similar to that in cells treated with CSF (DCX, 37.4%; Tuj-1, 42%) (Fig 6J, 6M, 6P and 6T and S3 Fig). BDNF treatment also increased the immunoreactivity of GFAP around neurospheres (29% *vs*. 8.5%, *P* <0.001) and the increase was similar between the BDNF and CSF (29.6%) treatments (Fig 6K, 6N, 6Q and 6U). In the time-lapse experiment, the migratory capacity of NSCs around neurospheres was higher after BDNF treatment (S3 Movie) than control treatment (S1 Movie), and was similar to that after CSF treatment (S2 Movie). Collectively, these findings point to the similarity between BDNF-induced and CSF-induced proliferation, differentiation (into neural and glial lineage cells), and migratory capacity of NSCs in cultured neurospheres.

### The expression of BDNF is increased at the SVZ in SAH rats

**Fig 7:**
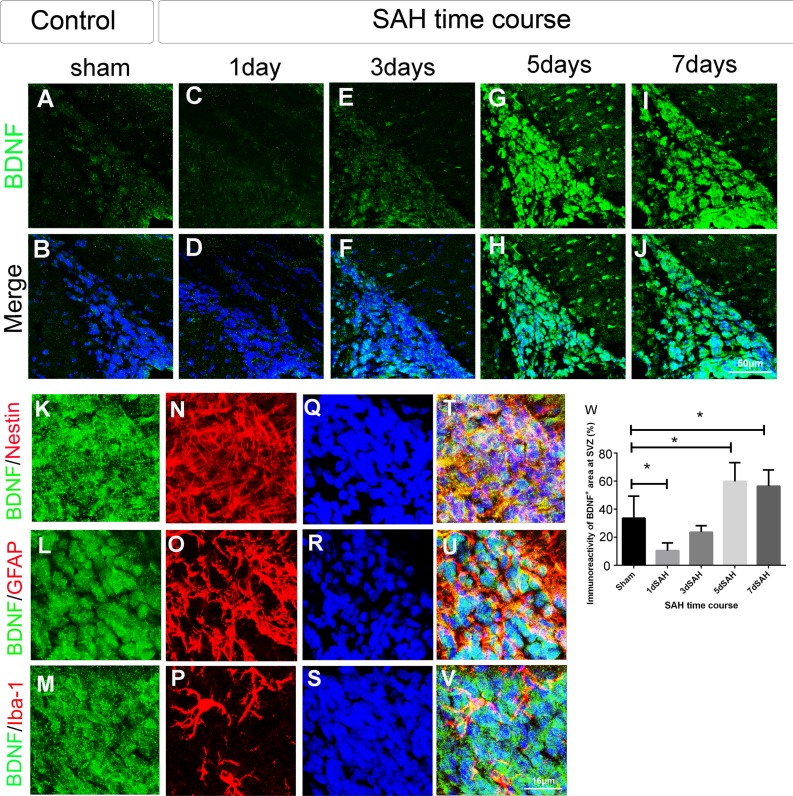
BDNF expression and its cellular localization in the SVZ of SAH rats. Coronal forebrain sections were immunostained with anti-BDNF, nestin, GFAP, Iba-1 (microglia) antibodies to determine the expression of BDNF after SAH. (A)-(J): photomicrograph showing the distribution of BDNF^+^ (A, C, E, G, and I) signal and as merged images (B, D, F, H, J) in the SVZ of animals with different SAH time course (1, 3, 5, and 7 days after SAH) and sham control. Scale bar = 50 μm. (K)-(V): Images showing BDNF^+^ (K, L and M), nestin^+^ (N), GFAP^+^ (O) and Iba-1^+^ (P) immunoreactivity and as merged images (T, U and V) on day 7 post SAH in the SVZ. Scale bar = 16 μm. (W): Percentage of BDNF^+^ area (BDNF^+^ area/ total area) in the SVZ. Means ± SD, n = 6 for each time-point. *, *P* < 0.05; one way ANOVA with Tukey's multiple comparisons test.

To demonstrate the expression of BDNF in the SVZ, forebrain sections from SAH rats at different post-SAH times were immunostained with anti-BDNF, anti-nestin, anti-GFAP, and anti-Iba-1 antibodies. BDNF immunoreactivity in the SVZ was significantly higher in SAH rats on post-SAH days 5 and 7 than in sham controls (59.8%, 56.4% *vs*. 33.6%, *P* < 0.05) (Fig 7A, 7G, 7I and 7W). At 7 days post SAH, nestin, GFAP, and Iba-1 were colocalized with BDNF (Fig 7K–7V), indicating that NSCs, astrocytes, and microglia all contributed to BDNF expression after SAH. Since microglia secrete trophic factors after activation [13], both anti-Iba-1 (a microglial marker) and anti-ED1 (an activated microglial marker) were used to assess microglial activation around SVZ. The immunoreactivities of Iba-1 and ED1 were both significantly higher in SAH rats on post-SAH days 3, 5, and 7 as compared to sham controls (Iba-1 33.8%, 40.6%, 47.2% *vs*. 17.2%, *P* < 0.05; ED1 6.9%, 10.9%, 15.5% *vs*. 2.5%, *P* < 0.01) (S4Y and S4Z Fig). The percentage of Iba-1-positive cells that are also positive for ED1 were increased on post-SAH days 3, 5, and 7 (17.8%, 28.8%, 40.5% *vs*. 16%, *P* < 0.01) (S4Aa Fig). The above results indicated that microglia are activated 3 to 7 days after SAH in the SVZ.

## Discussion

Adult neurogenesis is sensitive to pathologic stimuli, such as ischemic stroke and traumatic brain injury, which promote proliferation, differentiation, and migration of NSCs in the SVZ [14]. Investigating the post-insult endogenous neurogenesis might provide information needed for establishment of a future cell-based therapy. Although activation of endogenous neurogenesis after SAH has been noted in rodent and human brains [8, 9], the features and mechanisms of neurogenesis after SAH remain unclear. In the present study, neurogenesis activities including proliferation, differentiation, and migration of NSCs increased in the SVZ, 5 to 7 days after SAH. CSF collected from rats 5 to 7 days post SAH stimulated these activities in cultured NSCs, implying that certain factors in CSF could trigger neurogenesis. By ELISA analysis, we found that changes in BDNF concentration paralleled temporal changes in neurogenesis of NSCs after SAH. Both recombinant BDNF and CSF similarly enhanced neurogenesis in cultured NSCs. The expression of BDNF in the SVZ was also increased 5–7 days post SAH. These findings suggest that SAH enhances NSC proliferation, differentiation, and migration in the SVZ by upregulating BDNF expression.

After SAH, NSC proliferation in the SVZ declined in the first 3 days and then increased from days 5 to 7. The initial decline may result from a high concentration of free radicals derived from the degradation of hemoglobin and/or profound inflammation caused by the sudden increase of intracranial pressure [8, 15]. Notably, previous studies have shown that free radicals such as reactive oxygen or nitrogen species suppress SVZ neurogenesis by stimulating apoptosis or necrosis after cerebral infarct and traumatic brain injury [16, 17]. After transient suppression of NSC proliferation in the first few days post SAH, it may be the up-regulation of BDNF, which not only account for enhancement of NSC proliferation to a higher than pre-SAH level, but also increased NSC differentiation and migration in the SVZ [18–20].

The activity of neurogenesis is mainly mediated by growth factors and/or neurotrophic factors and growth/neurotrophic factors differ in their relationship to particular pathological conditions [21]. Among them, BDNF, NGF, EGF and EPO are important factors contributing to post-insult activation of neurogenesis [22–26]. In this study, although the concentrations of these factors all increased after SAH, only the temporal change in BDNF concentration paralleled the temporal change in NSC neurogenesis after SAH. In addition, recombinant BDNF at a concentration similar to that in CSF (7 days post SAH) enhanced the proliferation, differentiation, and migration of NSCs. BDNF is a potent modulator capable of regulating the functions of neurogenesis including neural regeneration and neuroprotection [27]. BDNF can also enhance synaptic efficacy and promote the formation of new synapses both in vitro and in vivo by morphological effects on dendrites and axons (a permissive mechanism) or promoting synapse formation directly (an instructive mechanism)[28, 29]. In this study, robust neurite sprouting demonstrated on day 5 and 7 post SAH both *in vivo* and *in vitro* matching the time course of NSC activation and BDNF upregulation suggests possible neuronal recovery and reconstruction of neural circuitry. Infusion or viral overexpression of BDNF in the cerebroventricles of adult rats can cause marked neurogenesis in the olfactory bulbs and striatum [20, 30–32]. Therefore, BDNF is likely the major neurotrophic factor triggering neurogenesis after SAH. There are still some limitations of this study regarding the detailed relationship between BDNF upregulation and the NSC stimulation. To further identified the role of BDNF in regulating neurogenesis after SAH, experiments utilizing BDNF inhibitor (BDNF scavenger, TrkB-Fc fusion protein or BDNF antibody, a specific inhibitor of Trk kinase activity, K252)[33–41] or knockout model (heterozygous BDNF knockout rats and in transgenic rats overexpressing truncated TrkB receptors)[42, 43] will be required. Furthermore, the results of the BDNF level in subacute phase of SAH, which was 7.87 pg/ml and 13.27 pg/ml on day 10 and 14 post SAH respectively (data not shown) indicating the expression of BDNF was downregulated compared to the level of BDNF on day 7 post SAH (793 pg/ml) and the neurogenesis may be regulated through other mechanisms in subacute phase of SAH. Additionally, the possibility that other growth factors or neurotrophic factors also play an independent or synergistic roles cannot be ruled out.

From post SAH day 5 to 7, the expression of BDNF increased in NSCs, astrocytes, and microglia in the SVZ and was especially high in NSCs on day 7 post SAH. BDNF from NSCs might further trigger neurogenesis in an autocrine and/or paracrine manner. Reacting to neural damage, astrocytes secrete many trophic factors such as BDNF [44–46]. Activated microglia under certain circumstances can be beneficial and support progenitor cell proliferation, survival, migration, and differentiation by secreting neurotrophic factors including BDNF [47–49]. The up-regulation of BDNF in astrocytes and microglia after SAH is likely also enhance activation of neurogenesis. However, the detailed relationship between these BDNF-expressing cells are unclear and may be complex. For example, previous studies have demonstrated that cytokines mediate the interaction between activated microglia and astrocytes and could be involved in the regulation of neurogenesis and neuroblast migration under pathological states [50, 51]. Whether SAH activates NSCs, astrocytes, and microglia simultaneously or sequentially to stimulate BDNF expression is unknown. In addition, the activation of microglia in acute inflammation (e.g., by bacterial endotoxin stimulation) is detrimental to NSCs proliferation and survival [52]. Whether microglial activation noted in this study has a predominantly beneficial or detrimental role to play in neurogenesis after SAH will require further study.

At present, there is no neuroprotective treatment for stroke, including subtypes of cerebral infarct, intracerebral hemorrhage, and SAH. Transgenic ablation of neurogenesis has been shown to impede motor function recovery after cerebral ischemia in mice, indicating the great importance of endogenous neurogenesis in the repair process following brain damage [53, 54]. In cerebral infarcts, NSCs in the SVZ proliferate and migrate to the infarct area and subsequently facilitate functional recovery via neurotrophic and cell-replacement effects [6, 7, 55]. In addition, gliogenesis may contribute to brain repair following brain injuries. In this study, we also demonstrated the proliferation, differentiation (into neural and glial lineage cells), and migration (to the striatum) were increased after SAH. The large amount of blood in subarachnoid space in SAH increases intracranial pressure, thereby causing inflammatory and toxic reactions to free radicals, which all potentially lead to neuronal death. The neurotrophic and cell-replacement effects of neurogenesis up-regulation after SAH might prevent cell death as noted in cerebral infarction. Although there is much remains to be done regarding the importance of neurogenesis activation in SAH and detailed relationship between BDNF upregulation and the NSC stimulation, the present study identified quantifiable and temporal features of neurogenesis in the SVZ that are necessary to guide future studies of SAH.

In summary, we demonstrated that neurogenesis (i.e., the proliferation, differentiation, and migration of NSCs) was enhanced on days 5 and 7 post SAH. This enhancement was likely mediated by up-regulation of BDNF expression in NSCs, astrocytes, and microglia of the SVZ. This study may provide a rationale for the use of BDNF as a targeted SAH therapy, considering no curative or neuroprotective therapy for SAH exists.

## Supporting Information

S1 Fig Post-SAH CSF promotes cell proliferation of cultured neurospheres at different concentrations. Primary cultured neurospheres treated with 0.25, 0.5, 1, and 2% CSF collected from rats on day 7 post SAH were immunostained with anti-nestin antibody (green: NSCs), anti-Ki67 antibody (red: proliferating cell) and Hoechst 33342 (blue: nucleus). (A)-(T): photomicrograph showing the distribution of Ki67^+^ (A, E, I, M, and Q) and nestin^+^ (B, F, J, N, and R) signals and as merged images (D, H, L, P, and T) in the neurospheres of CSF treatment or control. Scale bar = 50 μm. Images of Ki67^+^ /nestin^+^ neural stem cells in the neurospheres treated with 0.5% CSF showing Ki67 (U) and nestin (V) immunoreactivity separately or as merged image (X). Scale bar = 16 μm (Y): Percentage of proliferating cells (Ki67^+^ of Hoechst^+^ cells) in the neurospheres. Means ± SD, 12 neurospheres were counted for each concentration. ns: non-significant; ***, *P* < 0.001, one way ANOVA with Tukey's multiple comparisons test.
(TIF)

S2 Fig Treatment with post-SAH CSF enhances neuronal differentiation of cultured neurospheres. Neuronal differentiation were determined by immunocytostaining of cultured neurospheres with anti-Tuj-1 antibody (green: mature neuron) and Hoechst 33342 (blue: nucleus). (A)-(O): photomicrograph showing the distribution of Tuj-1^+^ (A, D, G, J, and M) signals and as merged images (C, F, I, L, and O) in the neurospheres treated with or without CSF. Scale bar = 50 μm. (P): Percentage of Tuj-1^+^ area (Tuj-1^+^ area/ total area). Means ± SD, 12 neurospheres were counted for each condition. ns: non-significant; ***, *P* < 0.001, one way ANOVA with Tukey's multiple comparisons test.
(TIF)

S3 Fig BDNF treatment enhances neuronal differentiation of cultured neurospheres. Neurospheres were immunostained with anti-Tuj-1 antibody (green: mature neuron) to determine the effects of recombinant BDNF (5 pg/ml) on neuronal differentiation of cultured neurospheres. The groups of neurospheres treated with 0.5% CSF (collected from rats on day 7 post SAH) and without treatment (control) were used for comparison. (A)-(I): photomicrograph showing the distribution of Tuj-1^+^ (A, D, and G) signals and as merged images with Hoechst 33342 (blue: nucleus) (C, F, and I). Scale bar = 50 μm. L (J): Percentage of Tuj-1^+^ area (Tuj-1^+^ area/ total area). Means ± SD, 12 neurospheres were counted for each condition. ns: non-significant; ***, *P* < 0.001, one way ANOVA with Tukey's multiple comparisons test.
(TIF)

S4 Fig Activation of microglia around the SVZ in SAH rats. Microglial activation in the SVZ was determined by double immunostaining of the forebrain sections with anti-Iba-1 antibody (green: microglia), ED-1 antibody (red: activated microglia), and Hoechst 33342 (blue: nucleus). (A)-(T): photomicrograph showing the distribution of Iba-1+ (A, E, I, M, and Q) and ED-1+ (B, F, J, N, and R) signals and as merged images (D, H, L, P, and T) in the SVZ of animals with different SAH time course (1, 3, 5, and 7 days after SAH) and sham control. Scale bar = 50 μm. (U)-(X): Images of ED-1+ /Iba-1+ activated microglia in the SVZ on day 7 post SAH showing Iba-1+ (U) and ED-1+ (V) immunoreactivity separately or as merged image (X). Scale bar = 16 μm. (Y): Percentage of Iba-1+ of Hoechst+ cells in the SVZ; (Z): Percentage of ED-1+ of Hoechst+ cells in the SVZ; (Aa) Percentage of activated microglia (ED1+ of Iba-1+ cells) in the SVZ. Means ± SD, n = 6 for each time-point. *, P < 0.05;**, P < 0.01; ***, P < 0.001, one way ANOVA with Dunnett's multiple comparisons test.
(TIF)

S1 Movie Cell migration around the cultured neurospheres using the time-lapse experiment. (MPG)

S2 Movie Cell migration around the cultured neurospheres using the time-lapse experiment. (MPG)

S3 Movie Cell migration around the cultured neurospheres using the time-lapse experiment. (MPG)

S1 Text Supporting Methods, including antibodies used in immunohistochemistry, antibodies used in immunocytochemistry, primary culture of neural stem cells, ELISA kits used in this experiments, and live cell imaging by time-lapse microscopy. (DOCX)

## References

[1] Rinkel GJ, Algra A. Long-term outcomes of patients with aneurysmal subarachnoid haemorrhage. Lancet Neurol 2011; 10: 349–356. 10.1016/S1474-4422(11)70017-5 21435599

[2] George PM, Steinberg GK. Novel stroke therapeutics: unraveling stroke pathophysiology and its impact on clinical treatments. Neuron 2015; 87: 297–309. 10.1016/j.neuron.2015.05.041 26182415 PMC4911814

[3] Doetsch F, García-Verdugo JM, Alvarez-Buylla A. Cellular composition and three-dimensional organization of the subventricular germinal zone in the adult mammalian brain. J Neurosci 1997; 17: 5046–5061. 9185542 10.1523/JNEUROSCI.17-13-05046.1997 PMC6573289

[4] Whitman MC, Greer CA. Adult neurogenesis and the olfactory system. Prog Neurobiol 2009; 89: 162–175. 10.1016/j.pneurobio.2009.07.003 19615423 PMC2748178

[5] Alvarez-Buylla A, Garcia-Verdugo JM. Neurogenesis in adult subventricular zone. J Neurosci 2002; 22: 629–634. 11826091 10.1523/JNEUROSCI.22-03-00629.2002 PMC6758521

[6] Thored P, Arvidsson A, Cacci E, Ahlenius H, Kallur T, Darsalia V, et al Persistent production of neurons from adult brain stem cells during recovery after stroke. Stem cells 2006; 24: 739–747. 10.1634/stemcells.2005-0281 16210404

[7] Arvidsson A, Collin T, Kirik D, Kokaia Z, Lindvall O. Neuronal replacement from endogenous precursors in the adult brain after stroke. Nat Med 2002; 8: 963–970. 10.1038/nm747 12161747

[8] Mino M, Kamii H, Fujimura M, Kondo T, Takasawa S, Okamoto H, et al Temporal changes of neurogenesis in the mouse hippocampus after experimental subarachnoid hemorrhage. Neurol Res 2003; 25: 839–845. 10.1179/016164103771953934 14669527

[9] Sgubin D, Aztiria E, Perin A, Longatti P, Leanza G. Activation of endogenous neural stem cells in the adult human brain following subarachnoid hemorrhage. J Neurosci Res 2007; 85: 1647–1655. 10.1002/jnr.21303 17455304

[10] Iliff JJ, Wang M, Liao Y, Plogg BA, Peng W, Gundersen GA, et al A paravascular pathway facilitates CSF flow through the brain parenchyma and the clearance of interstitial solutes, including amyloid β. Sci Transl Med 2012; 4: 147–159. 10.1126/scitranslmed.3003748 PMC3551275 22896675

[11] Suzuki SO, Goldman JE. Multiple cell populations in the early postnatal subventricular zone take distinct migratory pathways: a dynamic study of glial and neuronal progenitor migration. J Neurosci 2003; 23: 4240–4250. 12764112 10.1523/JNEUROSCI.23-10-04240.2003 PMC6741090

[12] Zhang RL, LeTourneau Y, Gregg SR, Wang Y, Toh Y, Robin AM, et al Neuroblast division during migration toward the ischemic striatum: a study of dynamic migratory and proliferative characteristics of neuroblasts from the subventricular zone. J Neurosci 2007; 27: 3157–3162. 10.1523/JNEUROSCI.4969-06.2007 17376977 PMC6672487

[13] Thored P, Heldmann U, Gomes-Leal W, Gisler R, Darsalia V, Taneera J, et al Long-term accumulation of microglia with proneurogenic phenotype concomitant with persistent neurogenesis in adult subventricular zone after stroke. Glia 2009; 57: 835–849. 10.1002/glia.20810 19053043

[14] Bellenchi GC, Volpicelli F, Piscopo V, Perrone-Capano C, di Porzio U. Adult neural stem cells: an endogenous tool to repair brain injury? J Neurochem 2013; 124: 159–167. 10.1111/jnc.12084 23134340

[15] Hayman LA, Pagani JJ, Kirkpatrick JB, Hinck VC. Pathophysiology of acute intracerebral and subarachnoid hemorrhage: applications to MR imaging. AJR Am J Roentgenol 1989; 153: 135–139. 10.2214/ajr.153.1.135 2660531

[16] Lewén A, Matz P, Chan PH. Free radical pathways in CNS injury. J Neurotrauma 2000; 17: 871–890. 11063054 10.1089/neu.2000.17.871

[17] Moreno-López B, Romero-Grimaldi C, Noval JA, Murillo-Carretero M, Matarredona ER, Estrada C. Nitric oxide is a physiological inhibitor of neurogenesis in the adult mouse subventricular zone and olfactory bulb. J Neurosci 2004; 24: 85–95. 10.1523/JNEUROSCI.1574-03.2004 14715941 PMC6729566

[18] Chen K, Henry RA, Hughes SM, Connor B. Creating a neurogenic environment: the role of BDNF and FGF2. Mol Cell Neurosci 2007; 36: 108–120. 10.1016/j.mcn.2007.06.004 17656107

[19] Kokaia Z, Zhao Q, Kokaia M, Elmér E, Metsis M, Smith ML, et al Regulation of brain-derived neurotrophic factor gene expression after transient middle cerebral artery occlusion with and without brain damage. Exp Neurol 1995; 136: 73–88. 10.1006/exnr.1995.1085 7589336

[20] Benraiss A, Chmielnicki E, Lerner K, Roh D, Goldman SA. Adenoviral brain-derived neurotrophic factor induces both neostriatal and olfactory neuronal recruitment from endogenous progenitor cells in the adult forebrain. J Neurosci 2001; 17: 6718–6731. 10.1523/JNEUROSCI.21-17-06718.2001 PMC6763117 11517261

[21] Christie KJ, Turnley AM. Regulation of endogenous neural stem/progenitor cells for neural repair-factors that promote neurogenesis and gliogenesis in the normal and damaged brain. Front Cell Neurosci 2013; 6: 1–18. 10.3389/fncel.2012.00070 PMC3548228 23346046

[22] Bath KG, Lee FS. Neurotrophic factor control of adult SVZ neurogenesis. Dev Neurobiol 2010; 70: 339–349. 10.1002/dneu.20781 20186714 PMC2917621

[23] Alagappan D, Lazzarino DA, Felling RJ, Balan M, Kotenko SV, Levison SW. Brain injury expands the numbers of neural stem cells and progenitors in the SVZ by enhancing their responsiveness to EGF. ASN Neuro 2009; 1: 95–111. 10.1042/AN20090002 PMC2695583 19570028

[24] Chiaramello S, Dalmasso G, Bezin L, Marcel D, Jourdan F, Peretto P, et al BDNF/ TrkB interaction regulates migration of SVZ precursor cells via PI3-K and MAP-K signalling pathways. Eur J Neurosci 2007; 26: 1780–1790. 10.1111/j.1460-9568.2007.05818.x 17883412

[25] Johanson C, Stopa E, Baird A, Sharma H. Traumatic brain injury and recovery mechanisms: peptide modulation of periventricular neurogenic regions by the choroid plexus-CSF nexus. J Neural Transm 2011; 118: 115–133. 10.1007/s00702-010-0498-0 20936524 PMC3026679

[26] Tsai PT, Ohab JJ, Kertesz N, Groszer M, Matter C, Gao J, et al A critical role of erythropoietin receptor in neurogenesis and post-stroke recovery. J Neurosci 2006; 26: 1269–1274. 10.1523/JNEUROSCI.4480-05.2006 16436614 PMC6674578

[27] Chen A, Xiong LJ, Tong Y, Mao M. The neuroprotective roles of BDNF in hypoxic ischemic brain injury. Biomed Rep 2013; 1: 167–176. 10.3892/br.2012.48 24648914 PMC3956206

[28] Bramham CR, Messaoudi E. BDNF function in adult synaptic plasticity: the synaptic consolidation hypothesis. Prog Neurobiol 2005; 76: 99–125. 10.1016/j.pneurobio.2005.06.003 16099088

[29] Ying Z, Roy RR, Zhong H, Zdunowski S, Edgerton VR, Gomez-Pinilla F. BDNF-exercise interactions in the recovery of symmetrical stepping after a cervical hemisection in rats. Neuroscience 2008; 155: 1070–1078. 10.1016/j.neuroscience.2008.06.057 18672032 PMC2805664

[30] Chmielnicki E, Benraiss A, Economides AN, Goldman SA. Adenovirally expressed noggin and brain-derived neurotrophic factor cooperate to induce new medium spiny neurons from resident progenitor cells in the adult striatal ventricular zone. J Neurosci 2004; 24: 2133–2142. 10.1523/JNEUROSCI.1554-03.2004 14999064 PMC6730416

[31] Henry RA, Hughes SM, Connor B. AAV-mediated delivery of BDNF augments neurogenesis in the normal and quinolinic acid-lesioned adult rat brain. Eur J Neurosci 2007; 25: 3513–3525. 10.1111/j.1460-9568.2007.05625.x 17610571

[32] Zigova T, Pencea V, Wiegand SJ, Luskin MB. Intraventricular administration of BDNF increases the number of newly generated neurons in the adult olfactory bulb. Mol Cell Neurosci 1998; 11: 234–245. 10.1006/mcne.1998.0684 9675054

[33] Shelton DL, Sutherland J, Gripp J, Camerato T, Armanini MP, Phillips HS, et al Human trks: molecular cloning, tissue distribution, and expression of extracellular domain immunoadhesins. J Neurosci 1995; 15: 477–491. 7823156 10.1523/JNEUROSCI.15-01-00477.1995 PMC6578290

[34] Lowenstein DH, Arsenault L. The effects of growth factors on the survival and differentiation of cultured dentate gyrus neurons. J Neurosci 1996; 16: 1759–1769. 8774444 10.1523/JNEUROSCI.16-05-01759.1996 PMC6578675

[35] Akaneya Y, Tsumoto T, Kinoshita S, Hatanaka H. Brain-derived neurotrophic factor enhances long-term potentiation in rat visual cortex. J Neurosci 1997; 17:6707–6716. 9254683 10.1523/JNEUROSCI.17-17-06707.1997 PMC6573135

[36] Kang H, Welcher AA, Shelton D, Schuman EM. Neurotrophins and time: different roles for TrkB signaling in hippocampal long-term potentiation. Neuron 1997; 19: 653–664. 9331355 10.1016/s0896-6273(00)80378-5

[37] Morrison ME, Mason CA. Granule neuron regulation of Purkinje cell development: striking a balance between neurotrophin and glutamate signaling. J Neurosci 1998; 18: 3563–3573. 9570788 10.1523/JNEUROSCI.18-10-03563.1998 PMC6793141

[38] Chen G, Kolbeck R, Barde YA, Bonhoeffer T, Kossel A. Relative contribution of endogenous neurotrophins in hippocampal long-term potentiation. J Neurosci 1999; 19: 7983–7990. 10479698 10.1523/JNEUROSCI.19-18-07983.1999 PMC6782442

[39] Numakawa T, Takei N, Hatanaka H. BDNF rapidly induces aspartate release from cultured CNS neurons. Neurosci Res 2000; 37: 59–65. 10802344 10.1016/s0168-0102(00)00103-6

[40] Ishikawa Y, Ikeuchi T, Hatanaka H. Brain-derived neurotrophic factor accelerates nitric oxide donor-induced apoptosis of cultured cortical neurons. J Neurochem 2000; 75: 494–502. 10899924 10.1046/j.1471-4159.2000.0750494.x

[41] Gratacos E, Checa N, Perez-Navarro E, Alberch J. Brain-derived neurotrophic factor (BDNF) mediates bone morphogenetic protein-2 (BMP-2) effects on cultured striatal neurones. J Neurochem 2001; 79: 747–755. 11723167 10.1046/j.1471-4159.2001.00570.x

[42] Kokaia M, Ernfors P, Kokaia Z, Elme´r E, Jaenisch R, Lindvall O. Suppressed epileptogenesis in BDNF mutant mice. Exp Neurol. 1995; 133: 215–224. 10.1006/exnr.1995.1024 7649227

[43] Lahteinen S, Pitkanen A, Saarelainen T, Nissinen J, Koponen E, Castren E. Decreased BDNF signalling in transgenic mice reduces epileptogenesis. Eur J Neurosci 2002; 15: 721–734. 11886452 10.1046/j.1460-9568.2002.01897.x

[44] Dougherty KD, Dreyfus CF, Black IB. Brain-derived neurotrophic factor in astrocytes, oligodendrocytes, and microglia/macrophages after spinal cord injury. Neurobiol Dis 2000; 7: 574–585. 10.1006/nbdi.2000.0318 11114257

[45] Fulmer CG, VonDran MW, Stillman AA, Huang Y, Hempstead BL, Dreyfus CF. Astrocyte-derived BDNF supports myelin protein synthesis after cuprizone-induced demyelination. J Neurosci 2014; 34: 8186–8196. 10.1523/JNEUROSCI.4267-13.2014 24920623 PMC4051974

[46] Saha RN, Liu X, Pahan K. Up-regulation of BDNF in astrocytes by TNF-alpha: a case for the neuroprotective role of cytokine. J Neuroimmune Pharmacol 2006; 1: 212–222. 10.1007/s11481-006-9020-8 18040799 PMC2131740

[47] Deierborg T, Roybon L, Inacio AR, Pesic J, Brundin P. Brain injury activates microglia that induce neural stem cell proliferation ex vivo and promote differentiation of neurosphere-derived cells into neurons and oligodendrocytes. Neuroscience 2010; 171: 1386–1396. 10.1016/j.neuroscience.2010.09.045 20883748

[48] Ekdahl CT, Kokaia Z, Lindvall O. Brain inflammation and adult neurogenesis: the dual role of microglia. Neuroscience 2009; 158: 1021–1029. 10.1016/j.neuroscience.2008.06.052 18662748

[49] Hanisch UK, Kettenmann H. Microglia: active sensor and versatile effector cells in the normal and pathologic brain. Nat Neurosci 2007; 10: 1387–1394. 10.1038/nn1997 17965659

[50] Das S, Basu A. Inflammation: a new candidate in modulating adult neurogenesis. J Neurosci Res 2008; 86: 1199–1208. 10.1002/jnr.21585 18058947

[51] Hanisch UK. Microglia as a source and target of cytokines. Glia 2002; 40: 140–155. 10.1002/glia.10161 12379902

[52] Monje ML, Toda H, Palmer TD. Inflammatory blockade restores adult hippocampal neurogenesis. Science 2003; 302: 1760–1765. 10.1126/science.1088417 14615545

[53] Jin K, Wang X, Xie L, Mao XO, Greenberg DA. Transgenic ablation of doublecortin-expressing cells suppresses adult neurogenesis and worsens stroke outcome in mice. Proc Natl Acad Sci USA 2010; 107: 7993–7998. 10.1073/pnas.1000154107 20385829 PMC2867852

[54] Sun F, Wang X, Mao X, Xie L, Jin K. Ablation of neurogenesis attenuates recovery of motor function after focal cerebral ischemia in middle-aged mice. PloS One 2012; 7: 1–8. 10.1371/journal.pone.0046326 PMC3482223 23110048

[55] Parent JM, Vexler ZS, Gong C, Derugin N, Ferriero DM. Rat forebrain neurogenesis and striatal neuron replacement after focal stroke. Ann Neurol 2002; 52: 802–813. 10.1002/ana.10393 12447935

