# Supplementary figures and images for: Subarachnoid Hemorrhage Promotes Proliferation, Differentiation, and Migration of Neural Stem Cells via BDNF Upregulation
Source: PLoS One. 2016 Nov 10;11(11):e0165460. doi: 10.1371/journal.pone.0165460 (PMC5104421; doi:10.1371/journal.pone.0165460)

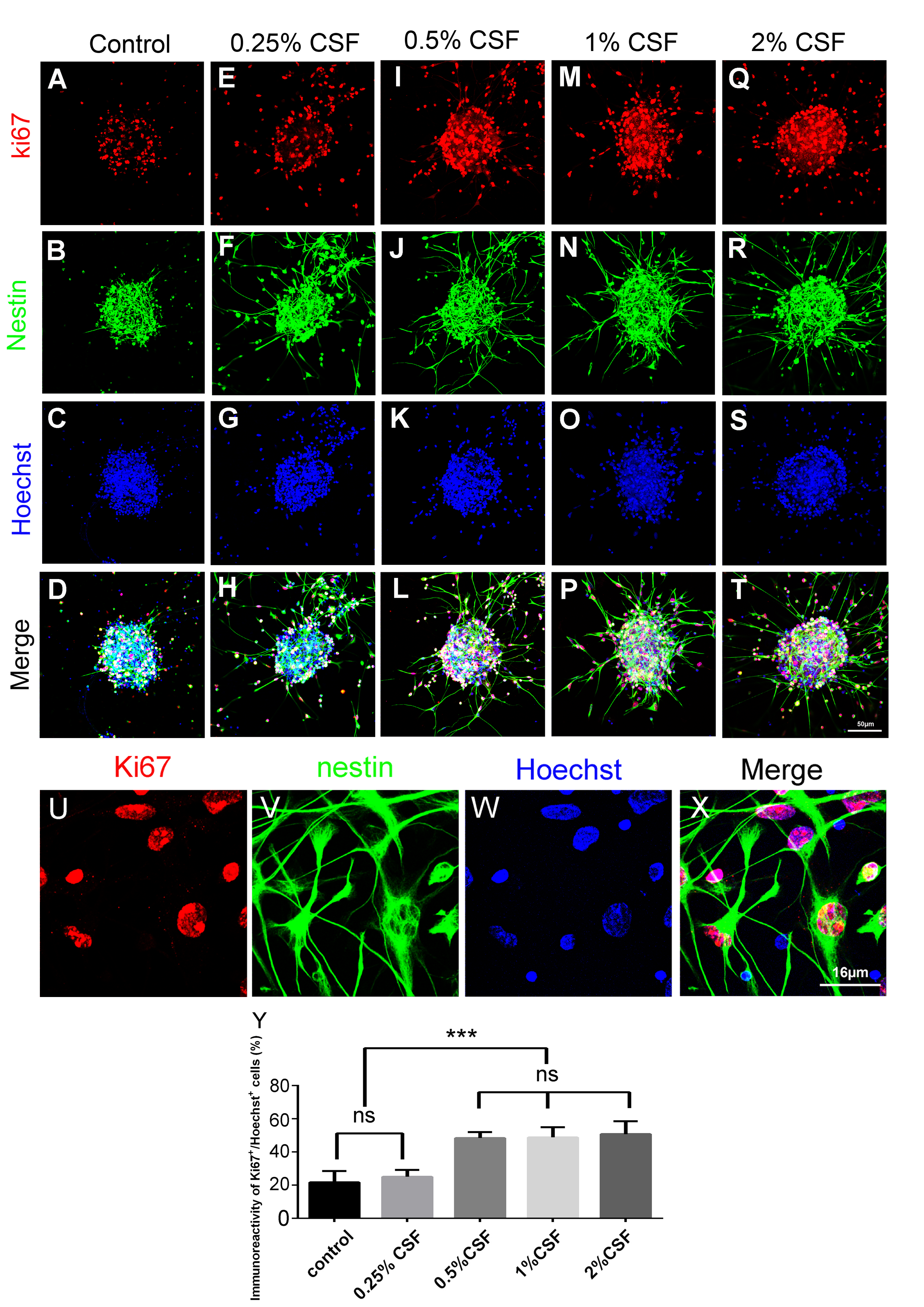

Supplement: S1 Fig — Primary cultured neurospheres treated with 0.25, 0.5, 1, and 2% CSF collected from rats on day 7 post SAH were immunostained with anti-nestin antibody (green: NSCs), anti-Ki67 antibody (red: proliferating cell) and Hoechst 33342 (blue: nucleus). (A)-(T): photomicrograph showing the distribution of Ki67+ (A, E, I, M, and Q) and nestin+ (B, F, J, N, and R) signals and as merged images (D, H, L, P, and T) in the neurospheres of CSF treatment or control. Scale bar = 50 μm. Images of Ki67+ /nestin+ neural stem cells in the neurospheres treated with 0.5% CSF showing Ki67 (U) and nestin (V) immunoreactivity separately or as merged image (X). Scale bar = 16 μm (Y): Percentage of proliferating cells (Ki67+ of Hoechst+ cells) in the neurospheres. Means ± SD, 12 neurospheres were counted for each concentration. ns: non-significant; ***, P < 0.001, one way ANOVA with Tukey's multiple comparisons test. (TIF) [file pone.0165460.s001.tif]

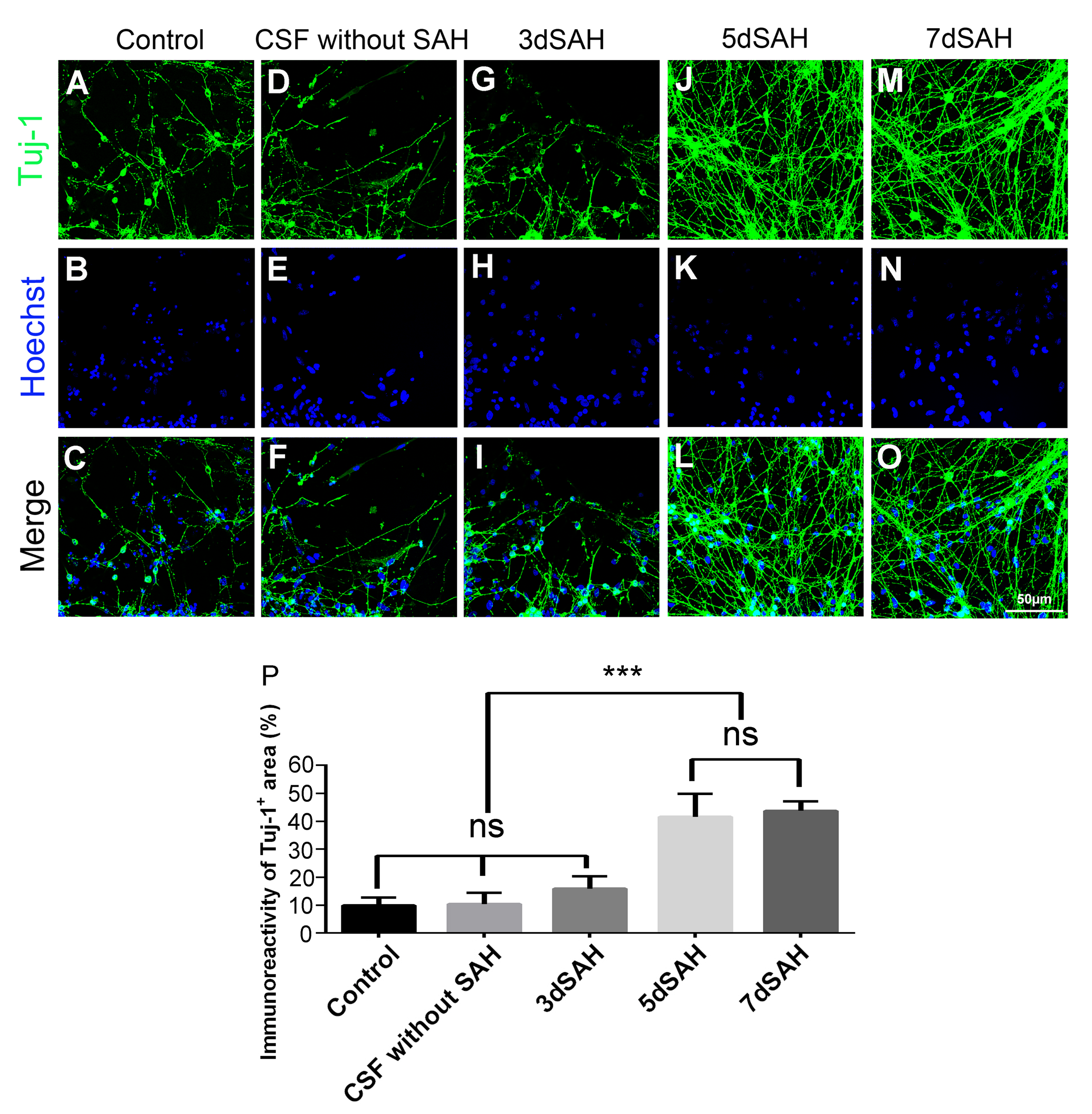

Supplement: S2 Fig — Neuronal differentiation were determined by immunocytostaining of cultured neurospheres with anti-Tuj-1 antibody (green: mature neuron) and Hoechst 33342 (blue: nucleus). (A)-(O): photomicrograph showing the distribution of Tuj-1+ (A, D, G, J, and M) signals and as merged images (C, F, I, L, and O) in the neurospheres treated with or without CSF. Scale bar = 50 μm. (P): Percentage of Tuj-1+ area (Tuj-1+ area/ total area). Means ± SD, 12 neurospheres were counted for each condition. ns: non-significant; ***, P < 0.001, one way ANOVA with Tukey's multiple comparisons test. (TIF) [file pone.0165460.s002.tif]

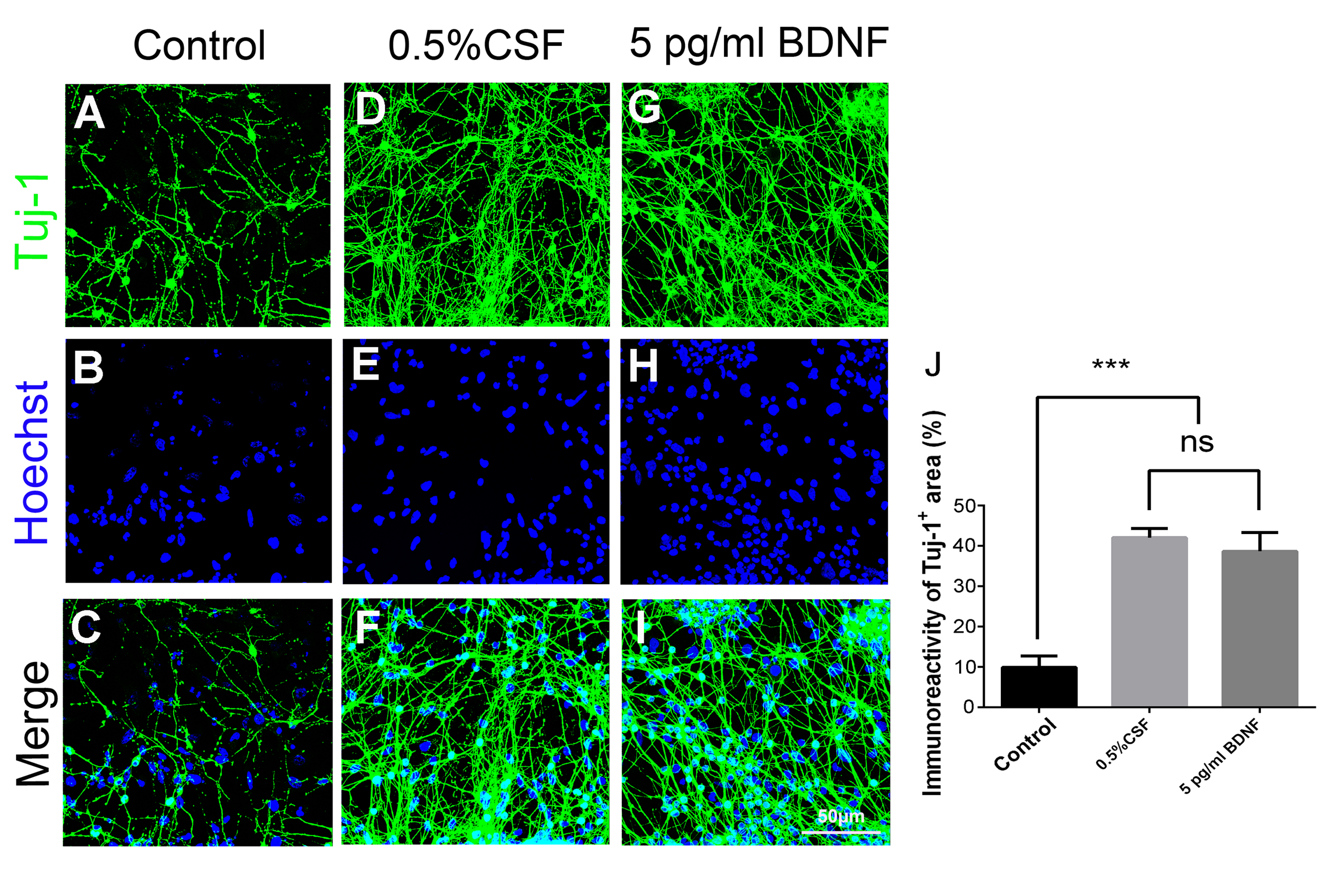

Supplement: S3 Fig — Neurospheres were immunostained with anti-Tuj-1 antibody (green: mature neuron) to determine the effects of recombinant BDNF (5 pg/ml) on neuronal differentiation of cultured neurospheres. The groups of neurospheres treated with 0.5% CSF (collected from rats on day 7 post SAH) and without treatment (control) were used for comparison. (A)-(I): photomicrograph showing the distribution of Tuj-1+ (A, D, and G) signals and as merged images with Hoechst 33342 (blue: nucleus) (C, F, and I). Scale bar = 50 μm. L (J): Percentage of Tuj-1+ area (Tuj-1+ area/ total area). Means ± SD, 12 neurospheres were counted for each condition. ns: non-significant; ***, P < 0.001, one way ANOVA with Tukey's multiple comparisons test. (TIF) [file pone.0165460.s003.tif]

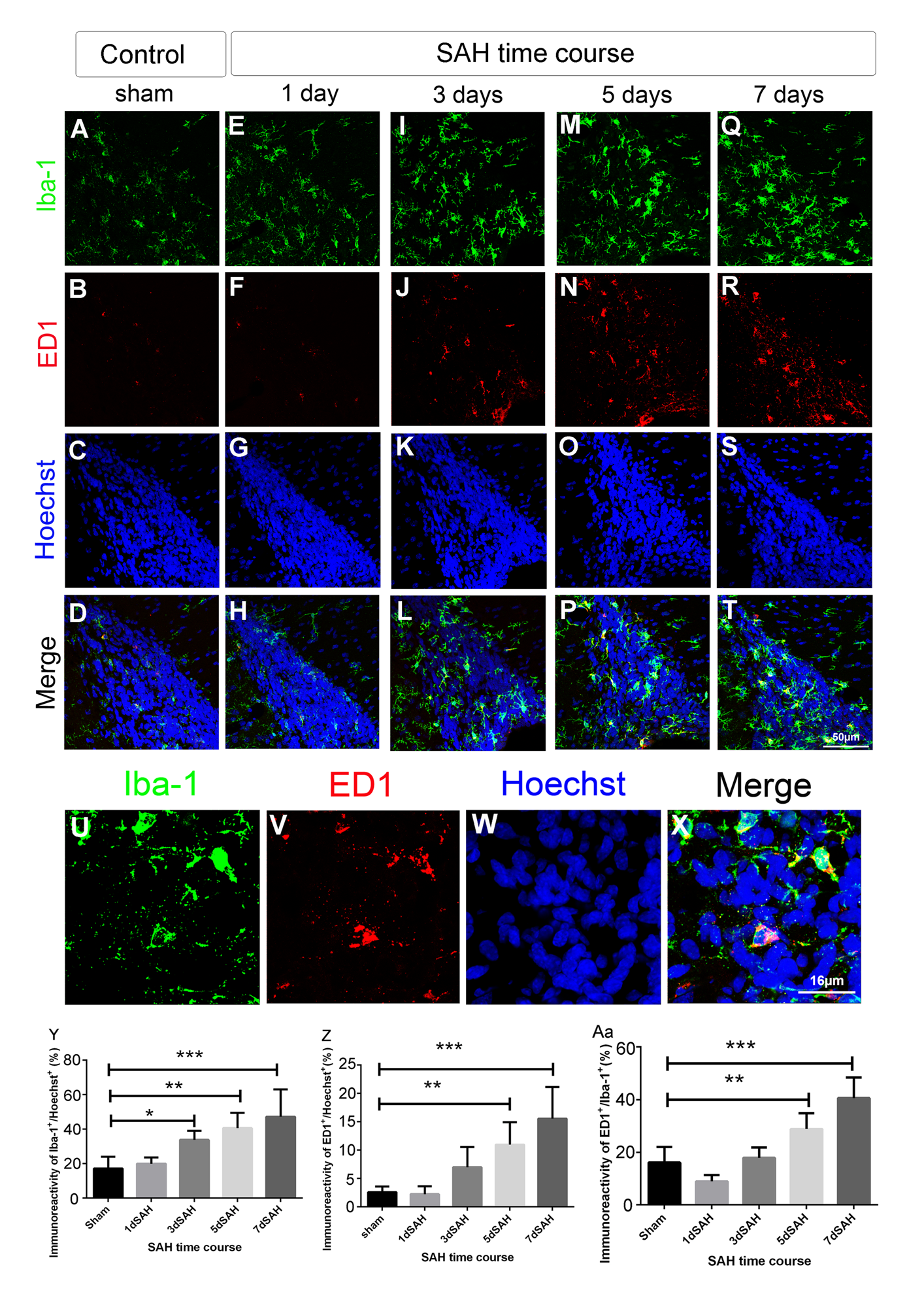

Supplement: S4 Fig — Microglial activation in the SVZ was determined by double immunostaining of the forebrain sections with anti-Iba-1 antibody (green: microglia), ED-1 antibody (red: activated microglia), and Hoechst 33342 (blue: nucleus). (A)-(T): photomicrograph showing the distribution of Iba-1+ (A, E, I, M, and Q) and ED-1+ (B, F, J, N, and R) signals and as merged images (D, H, L, P, and T) in the SVZ of animals with different SAH time course (1, 3, 5, and 7 days after SAH) and sham control. Scale bar = 50 μm. (U)-(X): Images of ED-1+ /Iba-1+ activated microglia in the SVZ on day 7 post SAH showing Iba-1+ (U) and ED-1+ (V) immunoreactivity separately or as merged image (X). Scale bar = 16 μm. (Y): Percentage of Iba-1+ of Hoechst+ cells in the SVZ; (Z): Percentage of ED-1+ of Hoechst+ cells in the SVZ; (Aa) Percentage of activated microglia (ED1+ of Iba-1+ cells) in the SVZ. Means ± SD, n = 6 for each time-point. *, P < 0.05;**, P < 0.01; ***, P < 0.001, one way ANOVA with Dunnett's multiple comparisons test. (TIF) [file pone.0165460.s004.tif]
