# Supplementary material for: Subarachnoid Hemorrhage Promotes Proliferation, Differentiation, and Migration of Neural Stem Cells via BDNF Upregulation
Source: PLoS One. 2016 Nov 10;11(11):e0165460. doi: 10.1371/journal.pone.0165460 (PMC5104421; doi:10.1371/journal.pone.0165460)
Supplement: S1 Text — (DOCX) [file pone.0165460.s008.docx]

**S1 Text**

**Antibodies used in immunohistochemistry**

Primary antibodies used in experiments included rabbit polyclonal anti-Ki67 (1:300, Catalog # ab15580, Abcam, Cambridge, MA); mouse monoclonal anti-nestin (1:1000, Catalog # ab6142, Abcam; 1:500, Catalog # 611659, BD Biosciences); rabbit polyclonal anti-doublecortin (DCX, 1:500, Catalog # ab18723, Abcam); mouse monoclonal anti-glial fibrillary acidic protein (GFAP, 1:200, Catalog # G3893, Sigma-Aldrich, St. Louis, MO); rabbit polyclonal anti-ionized calcium-binding adaptor molecule-1 (Iba-1, 1:500, Catalog # 019-19741, Wako Pure Chemicals Industries, Osaka, Japan); mouse monoclonal anti-Iba-1 (1:50, Catalog # MABN92, EMD Millipore, Billerica, MA); mouse monoclonal anti-ED1 (1:50, Catalog # MCA341GA, AbD serotec, Oxford, UK; 1:300, Catalog # MAB1435, EMD Millipore); rabbit polyclonal anti-BDNF (1:100, Catalog # AB1779SP, EMD Millipore). The secondary antibodies used in this experiment were Alexa 488 donkey anti-rabbit, Alexa 488 donkey anti-mouse, Alexa 594 donkey anti-rabbit, and Alexa 594 donkey anti-mouse (Molecular Probe, [Eugene](https://en.wikipedia.org/wiki/Eugene,_Oregon), OR) diluted 1:200.

**Antibodies used in immunocytochemistry**

Primary antibodies used in this experiment included rabbit polyclonal anti-Ki67 (1:500, Catalog # ab15580, Abcam); mouse monoclonal anti-nestin (1:200, Catalog # ab6142, Abcam); rabbit polyclonal anti-DCX (1:200, Catalog # ab18723, Abcam); mouse monoclonal anti-GFAP (1:300, Catalog # G3893, Sigma-Aldrich); mouse monoclonal anti-Tuj-1 (1:400, Catalog #MAB1637, EMD Millipore); chicken polyclonal anti-Tuj-1 (1:400, Catalog # AB9354, EMD Millipore). The secondary antibodies were Alexa 488 donkey anti-rabbit, Alexa 488 donkey anti-mouse, Alexa 594 donkey anti-rabbit and Alexa 594 donkey anti-mouse (Molecular Probe) diluted 1:200.

**Primary culture of neural stem cells**

Pregnant Wistar rats with gestational age of 15 days were sacrificed via intraperitoneal injection of an overdose of sodium pentobarbital. Embryos were removed from the amnion, and the telencephalon was dissected out and transferred to a 100-mm Petri dish containing 1% antibiotic-antimycotic (Life Technologies, Foster City, CA)-PBS solution. The telencephalon tissues were pooled in a 50-ml tube and centrifuged at 1000 rpm for 5 minutes. The supernatant was discarded and the tissue pellet was washed with PBS twice, resuspended in complete media containing Dulbecco’s Modified Eagle Media (DMEM)/F-12 (1:1) (Life Technologies) medium supplemented with 1% N2 supplement (Life Technologies), 20 ng/ml basic fibroblast growth factor (bFGF), and 1% antibiotic-antimycotic solution. The tissue pellet was then dissociated into single cells by triturating through a 1000-μl pipette. Cells were counted and seeded at a density of 2×10^6^ viable cells/cm^2^in T75 culture flasks (Corning, Corning, NY) containing 10 ml of complete media. Cultures were incubated at 37°C in a humidified atmosphere and 5% CO_2_ for 6 days by which time primary neurospheres had formed. Fresh medium and the growth factor were supplemented every two days.

**ELISA kits used in this experiments**

Mouse Erythropoietin (EPO) Quantikine ELISA Kit (R&D Systems, Minneapolis, MN); BDNF E_max_^®^ ImmunoAssay System (Promega, Madison, WI); Rat beta-neural growth factor (NGF) ELISA (RayBiotech, Norcross, GA); Rat epidermal growth factor (EGF) ELISA (RayBiotech).

**Live cell imaging by time-lapse microscopy**

Cells were seeded on Matrigel-coated coverslips at the density of 400 spheres per well in a 12-well plate and allowed to adhere for 4 hours before CSF treatment. Live cell images of neurospheres with different treatment were captured every 15 minutes for 24 hours by the Real-Time Cultured Cell Monitoring System CCM-1.4Z (ASTEC, Tokyo, Japan).
